# Novel Paramyxoviruses in Bats from Sub-Saharan Africa, 2007–2012

**DOI:** 10.3201/eid2110.140368

**Published:** 2015-10

**Authors:** Marinda Mortlock, Ivan V. Kuzmin, Jacqueline Weyer, Amy T. Gilbert, Bernard Agwanda, Charles E. Rupprecht, Louis H. Nel, Teresa Kearney, Jean M. Malekani, Wanda Markotter

**Affiliations:** University of Pretoria, Pretoria, South Africa (M. Mortlock, J. Weyer, L.H. Nel, W. Markotter);; University of Texas Medical Branch, Galveston, Texas, USA (I.V. Kuzmin);; National Institute for Communicable Diseases, Sandringham, South Africa (J. Weyer);; US Department of Agriculture, Fort Collins, Colorado, USA (A.T. Gilbert);; National Museums of Kenya, Nairobi, Kenya (B. Agwanda); LYSSA LLC, Atlanta, Georgia, USA (C.E. Rupprecht);; The Wistar Institute, Philadelphia, Pennsylvania, USA (C.E. Rupprecht);; Ditsong National Museum of Natural History, Pretoria (T. Kearney);; University of Kinshasa, Kinshasa, Democratic Republic of the Congo (J.M. Malekani)

**Keywords:** paramyxovirus, bats, Africa, epidemiology, emerging viruses, infectious diseases, zoonoses, bat-human interface, viruses, sub-Saharan Africa

## Abstract

As part of a larger survey for detection of pathogens among wildlife in sub-Saharan Africa conducted during 2007–2012, multiple diverse paramyxovirus sequences were detected in renal tissues of bats. Phylogenetic analysis supports the presence of at least 2 major viral lineages and suggests that paramyxoviruses are strongly associated with several bat genera.

Members of the *Paramyxoviridae* family are enveloped negative-sense RNA viruses, further classified into either the *Pneumovirinae* or *Paramyxovirinae* subfamily (*1*)*.*The *Paramyxovirinae* subfamily has increasingly been associated with bat species across the globe. The *Henipavirus* genus is 1 of 7 genera in this subfamily and contains the first recorded zoonotic paramyxoviruses, Hendra virus and Nipah virus. These 2 viruses are associated with severe respiratory and neurologic syndromes, and regular spillover from *Pteropus* spp. bats causes infections in humans and domestic animals (*2*). 

Enhanced surveillance for bat-associated pathogens has led to the discovery of numerous novel paramyxoviruses (*3**–**5*). *Henipavirus*-related viruses were identified in another pteropodid species, *Eidolon helvum*, sampled in Ghana, West Africa. This finding suggests an extension of the geographic and host ranges of the members of this virus genus (*6*)*.* Subsequent studies demonstrated a high diversity of paramyxoviruses in *E. helvum* bat population in Africa, as well as in other bat species from different continents. This finding suggests that bats may have a global role as potential paramyxovirus reservoirs (*3**,**4*). To contribute toward the knowledge of bat-associated paramyxovirus diversity and distribution, we sampled multiple bat species from several sub-Saharan African countries.

## The Study

During 2007–2012, we sampled 1,220 bats representing at least 48 species from multiple locations in selected countries in Africa (Table 1). Bats were anesthetized with the use of ketamine (0.05–0.1 mg/g body mass) and exsanguinated by cardiac puncture. Voucher specimens were identified through morphologic characterization (*7*) or, alternatively, through genetic barcoding. Approximately 30–100 mg of renal tissue was used for RNA extraction. A heminested primer set targeting the conserved polymerase (large) gene of *Respirovirus, Morbillivirus,* and *Henipavirus* was used for sample screening through reverse transcription PCR (*8*). A total of 103 samples (8.4%) tested positive, and the obtained amplicons of ≈490 bp were sequenced (Technical Appendix Table 1). For phylogenetic analysis, representative paramyxovirus sequences available from GenBank were included (Technical Appendix Table 2), and Bayesian analysis was performed by using BEAST version 1.7.4 software (http://beast.bio.ed.ac.uk/) (Figure).

**Table 1:** African bat species sampled and the number of paramyxovirus sequences detected in sub-Saharan Africa, by country, 2007–2012*

Southern Africa		
South Africa		
*Chaerephon ansorgei* (2/0)	***Neoromicia nana* (7/2)**	*Rhinolophus* sp. (1/0)
*Chaerephon pumilus* (8/0)	*Neoromicia rueppellii* (1/0)	*Rousettus aegyptiacus* (18/0)
*Epomophorus gambianus* (2/0)	*Neoromicia zuluensis* (1/0)	*Sauromys petrophilus* (1/0)
*Epomophorus wahlbergi* (15/0)	***Nycteris thebaica* (12/1)**	*Scotophilus* sp. (12/0)
***Eptesicus hottentotus* (2/1)**	*Nycticeinops schlieffeni* (9/0)	*Scotophilus dinganii* (26/0)
*Glauconycteris variegata* (5/0)	*Pipistrellus hesperidus* (5/0)	*Scotophilus leucogaster* (2/0)
***Hipposideros caffer* (6/2)**	*Pipistrellus rusticus* (5/0)	*Scotophilus nigrita* (1/0)
***Kerivoula argentata* (1/1)**	*Pipistrellus* sp. (5/0)	*Scotophilus viridis* (3/0)
*Miniopterus natalensis* (5/0)	*Rhinolophus darlingi* (5/0)	*Tadarida aegyptiaca* (5/0)
*Miniopterus* sp. (37/0)	***Rhinolophus denti* (3/2)**	*Taphozous mauritianus* (2/0)
*Mops condylurus* (7/0)	*Rhinolophus fumigatus* (2/0)	
*Neoromicia capensis* (16/0)	***Rhinolophus landeri* (1/1)**	
*Neoromicia helios* (6/0)	*Rhinolophus simulator* (2/0)	
Swaziland		
*Nycteris thebaica* (4/0)		
Eastern Africa		
Kenya		
*Coleura afra* (27/10)	*Miniopterus natalensis* (15/0)	***Rousettus aegyptiacus (84/2)***
*Eidolon helvum* (15/0)	***Miniopterus* sp. (77/13)**	*Scotoecus* sp. (2/0)
*Epomophorus labiatus* (6/0)	*Neoromicia* sp. (25/0)	*Scotophilus dinganii* (2/0)
*Epomophorus wahlbergi* (2/0)	***Nycteris* sp. (2/1)**	*Taphozous* sp. (1/0)
*Hipposideros vittatus* (71/0)	***Otomops martiensseni* (40/9)**	***Triaenops afer* (16/12)**
***Hipposideros* sp. (8/1)**	*Rhinolophus landeri* (12/0)	
***Miniopterus minor* (151/14)**	*Rhinolophus* sp. (14/0)	
Central Africa		
Cameroon		
*Chaerephon* sp. (32/0)	***Hipposideros* sp. (39/1)**	***Taphozous* sp. (12/3)**
*Eidolon helvum* (15/0)	***Rhinolophus* sp. (9/1)**	
*Epomophorus* sp. (1/0)	*Scotophilus dinganii* (1/0)	
Democratic Republic of the Congo		
*Chaerephon pumilus* (25/0)	*Hypsignathus monstrosus* (2/0)	*Myonycteris torquata* (8/0)
*Chaerephon* sp. (22/0)	*Megaloglossus woermanni* (10/0)	*Myotis* sp. (3/0)
*Eidolon helvum* (22/0)	*Micropteropus pusillus* (1/0)	*Neoromicia* sp. (1/0)
*Glauconycteris argentata* (1/0)	*Mimetillus moloneyi* (1/0)	***Pipistrellus* sp. (40/20)**
***Hipposideros fuliginosus* (21/3)**	***Miniopterus* sp. (41/2)**	*Rhinolophus* sp. (1/0)
*Hipposideros gigas* (2/0)	*Mops condylurus* (33/0)	*Scotophilus dinganii* (2/0)
Western Africa		
Nigeria		
*Eidolon helvum* (20/0)	***Hipposideros* sp. (3/1)**	*Rousettus aegyptiacus* (21/0)
*Hipposideros vittatus* (8/0)	*Lissonycteris angolensis* (8/0)	

*Values are no. samples (no. positive). Boldface indicates implicated species. The sampling protocol was approved by the Institutional Animal Care and Use Committee of the Centers for Disease Control and Prevention; protocol 2096FRAMULX-A3 and The University of Pretoria Animal Ethics Committee (EC054–14).

**Figure:**
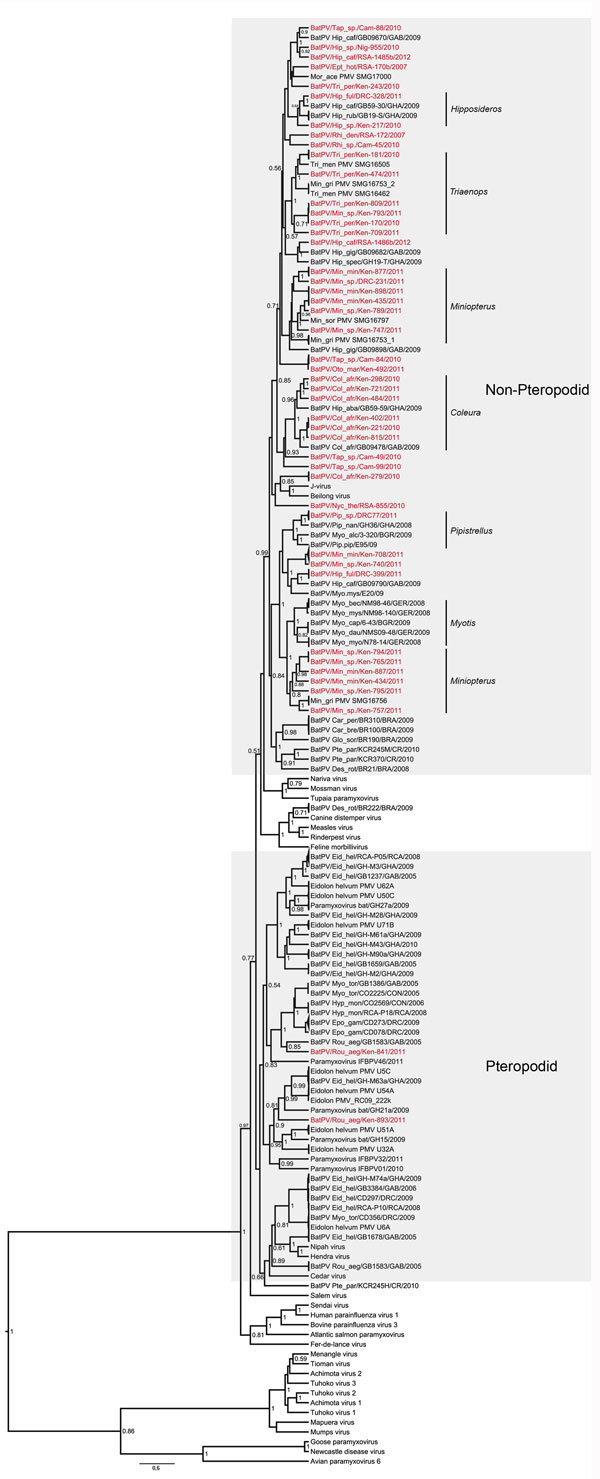
Maximum clade credibility tree based on partial polymerase (large) gene sequences (439 bp) of paramyxoviruses built in BEAST version 1.7.4 software (http://beast.bio.ed.ac.uk/), applying the general time reversible plus invariant sites plus gamma model inferred by jModelTest version 0.1.1 (*10*). Sequences detected in this study are indicated in red. Identical sequences were collapsed to only show a representative. Genus-specific clusters are indicated on the right and show possible opportunistic infections in other species grouping within these clusters. The 2 lineages reported are indicated in the gray areas.

Several samples from bat species not previously implicated as paramyxovirus reservoirs tested positive in our study. Some of these implicated species are known to roost in peridomestic environments. Sequence analysis of paramyxovirus sequences showed a clear bifurcation of the phylogenetic tree, segregating paramyxoviruses detected in pteropodid bats (Pteropodidae) from paramyxoviruses detected in bats of other families (Figure). The former contained henipaviruses and related viruses. Two viral sequences detected in *Rousettus aegyptiacus* bats grouped within this cluster as part of a sister clade to the henipaviruses. The second cluster contained sequences derived from nonpteropodid bats. Some of these sequences grouped with the sequences from the *Morbillivirus* and proposed *Jeilongvirus* genera, whereas others could not be included in any of the other paramyxovirus genera.

We observed a strong association of several viral lineages to particular bat genera for paramyxoviruses identified in *Hipposideros*, *Miniopterus*, *Coleura*, *Myotis,* and *Pipistrellus* bats, although the bats were sampled from geographically distant locations. In contrast to the sequences of European and South American origin, for which geographic clustering was observed, no such clustering was found among the sequences from African bats.

The incidence and diversity of viral sequences varied according to bat species. For example, nearly identical sequences were detected in 50% of *Pipistrellus* spp. sampled from a single colony in the Democratic Republic of the Congo (n = 40). In other cases, several distinct viral sequences were detected in different individual bats of 1 species, such as *Miniopterus minor* bats sampled from a single colony in Kenya (n = 53), which harbored 6 distinct viral sequences. Some of the sequences were found more frequently than others. In contrast to a previous study which did not identify paramyxoviruses in *Coleura afra* bats sampled in Ghana (n = 71) (*4*), we detected a substantial paramyxovirus incidence (37%, n = 27) in the same bat species sampled in Kenya (Table 2).

**Table 2:** Paramyxovirus incidence in selected bat species from various African countries*

Species	Country	Tissue type†	No. sampled	No. positive	Incidence, %	Reference
*Coleura afra*	Ghana	‡	71	0	0.0	(*4*)
	Kenya	Kidney	27	10	37.0	§
	Central Africa¶	Spleen	25	1	4.0	(*4*)
*Eidolon helvum*	Cameroon	Kidney	15	0	0.0	§
	DRC	Kidney	22	0	0.0	§
	Ghana	All solid organs, blood	673	67	10.0	(*4*)
	Kenya	Kidney	15	0	0.0	§
	Central Africa	Spleen	49	17	34.5	(*4*)
	Nigeria	Kidney	20	0	0.0	§
	Republic of Congo	All solid organs, blood, salivary gland, throat swab, feces, urine	42	11	26.2	(*9*)
*Epomophorus gambianus*	Central Africa	Spleen	48	3	6.3	(*4*)
	South Africa	Kidney	2	0	0.0	§
	Ghana	‡	20	1	5.0	(*4*)
*Hipposideros caffer*	Central Africa	Spleen	337	3	0.9	(*4*)
	South Africa	Kidney	6	2	33.3	§
	DRC	Kidney	2	0	0.0	§
*Hipposideros gigas*	Gabon	Spleen	196	3	1.5	(*4*)
	DRC	Kidney	2	0	0.0	§
*Hypsignathus monstrosus*	Central Africa	Spleen	53	4	7.5	(*4*)
	DRC	Kidney	10	0	0.0	§
*Megaloglossus woermanni*	Central Africa	Spleen	34	1	2.9	(*4*)
	DRC	Kidney	8	0	0.0	§
*Myonycteris torquata*	Central Africa	Spleen	111	3	2.7	(*4*)
	Ghana	‡	1	0	0.0	(*4*)
*Rhinolophus landeri*	Kenya	Kidney	12	0	0.0	§
	South Africa	Kidney	1	1	100.0	§
	Ghana	‡	30	0	0.0	(*4*)
*Rousettus aegyptiacus*	Kenya	Kidney	84	2	2.4	§
	Central Africa	Spleen	183	18	9.8	(*4*)
	Nigeria	Kidney	21	0	0.0	§
	South Africa	Kidney	18	0	0.0	§

*DRC, Democratic Republic of the Congo. †Tissue type stated for positive samples only and may not indicate all tissues sampled. ‡Information not available. §Species and countries sampled during this study. ¶Central Africa refers to Gabon/Republic of Congo/DRC/Republic of Central Africa.

## Conclusions

The henipaviruses were the first bat paramyxoviruses directly linked to human disease; however, most aspects of pathogenicity and the host ranges of the increasingly detected novel bat paramyxoviruses remain to be investigated. Here we report information regarding paramyxovirus distribution through molecular evidence of bat-associated paramyxoviruses in Cameroon, Nigeria, and South Africa, as well as evidence of paramyxoviruses in nonpteropodid bats from the Democratic Republic of the Congo. Our results suggest that 2 separate lineages were established during the evolution of bat-associated paramyxoviruses: the pteropodid bats potentially harbor 1 lineage, and the nonpteropodid bats potentially harbor the other. In contrast to the proposed chiropteran classification, which supports a sister-taxon relationship between Rhinolophoidae and Pteropodidae on the suborder level, paramyxovirus divergence appears to correlate with traditional bat taxonomy. The evolution behind this divergence might be a result of multiple evolutionary origins or a single origin with subsequent divergence. As with the evolution of echolocation, this question remains to be answered (*11*). More extensive bat sampling and molecular dating of the paramyxovirus phylogeny may help resolve this question.

Intensified anthropogenic transformations have facilitated closer contact between humans, domestic animal populations, and wildlife. Our study demonstrates that some bat species, adapted to peridomestic roosting, can have a substantial incidence of diverse paramyxoviruses. The variation in incidence and viral diversity observed in several bat species may suggest that some species are the true reservoirs, whereas others are mere incidental hosts. Given the observed virus diversity, implications for public health and veterinary medicine should be taken into account, especially considering the known likelihood of direct bat-to-human and human-to-human transmission of Nipah virus (*12*). Enhanced surveillance in bats and other animals will be useful for detecting possible spillover events and host shifts. Clearly, systematic longitudinal studies are needed to elucidate critical factors of paramyxovirus circulation within bat communities (*13*), and further research is needed to clarify the pathobiology, tissue tropism, and excretion pathways of these novel paramyxoviruses because these factors can be directly related to their zoonotic potential.

Technical Appendix Paramyxovirus sequences detected in this study
