# Supplementary material for: Novel Paramyxoviruses in Bats from Sub-Saharan Africa, 2007–2012
Source: Emerg Infect Dis. 2015 Oct;21(10):1840–3. doi: 10.3201/eid2110.140368 (PMC4593419; doi:10.3201/eid2110.140368)
Supplement: Technical Appendix — Paramyxovirus sequences detected in this study [file 14-0368-Techapp-s1.pdf]

Article DOI: http://dx.doi./org/10.3201/eid2110.140368

Novel Paramyxoviruses in Bats from Sub-Saharan Africa, 2007–2012

Technical Appendix

Technical Appendix Table 1. Paramyxovirus sequences detected in this study

Sample	Country	Year	Bat species	GenBank sequence name	Accession number
Cam-45	Cameroon	2010	*Rhinolophus* sp.	BatPV/Rhi_sp./Cam-45/2010	KC578678
Cam-49	Cameroon	2010	*Taphozous* sp.	BatPV/Tap_sp./Cam-49/2010	KC578679
Cam-84	Cameroon	2010	*Taphozous* sp.	BatPV/Tap_sp./Cam-84/2010	KC578680
Cam-88	Cameroon	2010	*Hipposideros* sp.	BatPV/Hip_sp./Cam-88/2010	KC578681
Cam-99	Cameroon	2010	*Taphozous* sp.	BatPV/Tap_sp./Cam-99/2010	KC578682
DRC-04	DRC	2011	*Pipistrellus* sp.	BatPV/Pip_sp./DRC-04/2011	KC578653
DRC-08	DRC	2011	*Pipistrellus* sp.	BatPV/Pip_sp./DRC-08/2011	KC578654
DRC-09	DRC	2011	*Pipistrellus* sp.	BatPV/Pip_sp./DRC-09/2011	KC578655
DRC-10	DRC	2011	*Pipistrellus* sp.	BatPV/Pip_sp./DRC-10/2011	KC578656
DRC-11	DRC	2011	*Pipistrellus* sp.	BatPV/Pip_sp./DRC-11/2011	KC578657
DRC-51	DRC	2011	*Pipistrellus* sp.	BatPV/Pip_sp./DRC-51/2011	KC578658
DRC-54	DRC	2011	*Pipistrellus* sp.	BatPV/Pip_sp./DRC-54/2011	KC578659
DRC-72	DRC	2011	*Pipistrellus* sp.	BatPV/Pip_sp./DRC-72/2011	KC578660
DRC-75	DRC	2011	*Pipistrellus* sp.	BatPV/Pip_sp./DRC75/2011	KC578661
DRC-77	DRC	2011	*Pipistrellus* sp.	BatPV/Pip_sp./DRC77/2011	KC578662
DRC-79	DRC	2011	*Pipistrellus* sp.	BatPV/Pip_sp./DRC-79/2011	KC578663
DRC-82	DRC	2011	*Pipistrellus* sp.	BatPV/Pip_sp./DRC-82/2011	KC578664
DRC-83	DRC	2011	*Pipistrellus* sp.	BatPV/Pip_sp./DRC-83/2011	KC578665
DRC-85	DRC	2011	*Pipistrellus* sp.	BatPV/Pip_sp./DRC-85/2011	KC578666
DRC-86	DRC	2011	*Pipistrellus* sp.	BatPV/Pip_sp./DRC-86/2011	KC578667
DRC-90	DRC	2011	*Pipistrellus* sp.	BatPV/Pip_sp./DRC-90/2011	KC578668
DRC-92	DRC	2011	*Pipistrellus* sp.	BatPV/Pip_sp./DRC-92/2011	KC578669
DRC-94	DRC	2011	*Pipistrellus* sp.	BatPV/Pip_sp./DRC-94/2011	KC578670
DRC-112	DRC	2011	*Pipistrellus* sp.	BatPV/Pip_sp./DRC-112/2011	KC578671
DRC-113	DRC	2011	*Pipistrellus* sp.	BatPV/Pip_sp./DRC-113/2011	KC578672
DRC-216	DRC	2011	*Miniopterus* sp.	BatPV/Min_sp./DRC-216/2011	KC578673
DRC-231	DRC	2011	*Miniopterus* sp.	BatPV/Min_sp./DRC-231/2011	KC578674
DRC-328	DRC	2011	*Hipposideros fuliginosus*	BatPV/Hip_ful/DRC-328/2011	KC578675
DRC-388	DRC	2011	*Hipposideros fuliginosus*	BatPV/Hip_ful/DRC-388/2011	KC578676
DRC-399	DRC	2011	*Hipposideros fuliginosus*	BatPV/Hip_ful/DRC-399/2011	KC578677
Ken-170	Kenya	2010	*Triaenops afer*	BatPV/Tri_per/Ken-170/2010	KC578640
Ken-181	Kenya	2010	*Triaenops afer*	BatPV/Tri_per/Ken-181/2010	KC578641
Ken-217	Kenya	2010	*Hipposideros* sp.	BatPV/Hip_sp./Ken-217/2010	KC578642
Ken-219	Kenya	2010	*Coleura afra*	BatPV/Col_afr/Ken-219/2010	KC578643
Ken-221	Kenya	2010	*Coleura afra*	BatPV/Col_afr/Ken-221/2010	KC578644
Ken-241	Kenya	2010	*Triaenops afer*	BatPV/Tri_per/Ken-241/2010	KC578645
Ken-243	Kenya	2010	*Triaenops afer*	BatPV/Tri_per/Ken-243/2010	KC578646
Ken-279	Kenya	2010	*Coleura afra*	BatPV/Col_afr/Ken-279/2010	KC578647
Ken-292	Kenya	2010	*Triaenops afer*	BatPV/Tri_per/Ken-292/2010	KC578648
Ken-298	Kenya	2010	*Coleura afra*	BatPV/Col_afr/Ken-298/2010	KC578649
Ken-300	Kenya	2010	*Triaenops afer*	BatPV/Tri_per/Ken-300/2010	KC578650
Ken-345	Kenya	2011	*Miniopterus minor*	BatPV/Min_min/Ken-345/2011	KC578591
Ken-355	Kenya	2011	*Otomops martiensseni*	BatPV/Oto_mar/Ken-355/2011	KC578592
Ken-402	Kenya	2011	*Coleura afra*	BatPV/Col_afr/Ken-402/2011	KC578593
Ken-412	Kenya	2011	*Miniopterus minor*	BatPV/Min_min/Ken-412/2011	KC578594
Ken-414	Kenya	2011	*Miniopterus minor*	BatPV/Min_min/Ken-414/2011	KC578595
Ken-415	Kenya	2011	*Miniopterus minor*	BatPV/Min_min/Ken-415/2011	KC578596
Ken-434	Kenya	2011	*Miniopterus minor*	BatPV/Min_min/Ken-434/2011	KC578597
Ken-435	Kenya	2011	*Miniopterus minor*	BatPV/Min_min/Ken-435/2011	KC578598
Ken-438	Kenya	2011	*Otomops martiensseni*	BatPV/Oto_mar/Ken-438/2011	KC578599
Ken-439	Kenya	2011	*Otomops martiensseni*	BatPV/Oto_mar/Ken-439/2011	KC578600
Ken-462	Kenya	2011	*Otomops martiensseni*	BatPV/Oto_mar/Ken-462/2011	KC578601
Ken-474	Kenya	2011	*Triaenops afer*	BatPV/Tri_per/Ken-474/2011	KC578602

Sample	Country	Year	Bat species	GenBank sequence name	Accession number
Ken-484	Kenya	2011	*Coleura afra*	BatPV/Col_afr/Ken-484/2011	KC578603
Ken-490	Kenya	2011	*Otomops martiensseni*	BatPV/Oto_mar/Ken-490/2011	KC578604
Ken-491	Kenya	2011	*Otomops martiensseni*	BatPV/Oto_mar/Ken-491/2011	KC578605
Ken-492	Kenya	2011	*Otomops martiensseni*	BatPV/Oto_mar/Ken-492/2011	KC578606
Ken-514	Kenya	2010	*Otomops martiensseni*	BatPV/Oto_mar/Ken-514/2010	KC578651
Ken-534	Kenya	2010	*Otomops martiensseni*	BatPV/Oto_mar/Ken-534/2010	KC578652
Ken-678	Kenya	2011	*Miniopterus minor*	BatPV/Min_min/Ken-678/2011	KC578607
Ken-681	Kenya	2011	*Triaenops afer*	BatPV/Tri_per/Ken-681/2011	KC578608
Ken-708	Kenya	2011	*Miniopterus minor*	BatPV/Min_min/Ken-708/2011	KC578609
Ken-709	Kenya	2011	*Triaenops afer*	BatPV/Tri_per/Ken-709/2011	KC578610
Ken-712	Kenya	2011	*Nycteris* sp.	BatPV/Nyc_sp./Ken-712/2011	KC578611
Ken-718	Kenya	2011	*Triaenops afer*	BatPV/Tri_per/Ken-718/2011	KC578612
Ken-721	Kenya	2011	*Coleura afra*	BatPV/Col_afr/Ken-721/2011	KC578613
Ken-740	Kenya	2011	*Miniopterus* sp.	BatPV/Min_sp./Ken-740/2011	KC578614
Ken-747	Kenya	2011	*Miniopterus* sp.	BatPV/Min_sp./Ken-747/2011	KC578615
Ken-756	Kenya	2011	*Miniopterus* sp.	BatPV/Min_sp./Ken-756/2011	KC578616
Ken-757	Kenya	2011	*Miniopterus* sp.	BatPV/Min_sp./Ken-757/2011	KC578617
Ken-765	Kenya	2011	*Miniopterus* sp.	BatPV/Min_sp./Ken-765/2011	KC578618
Ken-766	Kenya	2011	*Miniopterus* sp.	BatPV/Min_sp./Ken-766/2011	KC578619
Ken-769	Kenya	2011	*Miniopterus* sp.	BatPV/Min_sp./Ken-769/2011	KC578620
Ken-776	Kenya	2011	*Miniopterus* sp.	BatPV/Min_sp./Ken-776/2011	KC578621
Ken-787	Kenya	2011	*Miniopterus* sp.	BatPV/Min_sp./Ken-787/2011	KC578622
Ken-789	Kenya	2011	*Miniopterus* sp.	BatPV/Min_sp./Ken-789/2011	KC578623
Ken-793	Kenya	2011	*Miniopterus* sp.	BatPV/Min_sp./Ken-793/2011	KC578624
Ken-794	Kenya	2011	*Miniopterus* sp.	BatPV/Min_sp./Ken-794/2011	KC578625
Ken-795	Kenya	2011	*Miniopterus* sp.	BatPV/Min_sp./Ken-795/2011	KC578626
Ken-803	Kenya	2011	*Miniopterus minor*	BatPV/Min_min/Ken-803/2011	KC578627
Ken-804	Kenya	2011	*Miniopterus minor*	BatPV/Min_min/Ken-804/2011	KC578628
Ken-808	Kenya	2011	*Triaenops afer*	BatPV/Tri_per/Ken-808/2011	KC578629
Ken-809	Kenya	2011	*Triaenops afer*	BatPV/Tri_per/Ken-809/2011	KC578630
Ken-814	Kenya	2011	*Coleura afra*	BatPV/Col_afr/Ken-814/2011	KC578631
Ken-815	Kenya	2011	*Coleura afra*	BatPV/Col_afr/Ken-815/2011	KC578632
Ken-839	Kenya	2011	*Rousettus aegyptiacus*	BatPV/Rou_aeg/Ken-893/2011	KC578633
Ken-841	Kenya	2011	*Rousettus aegyptiacus*	BatPV/Rou_aeg/Ken-841/2011	KC578634
Ken-856	Kenya	2011	*Coleura afra*	BatPV/Col_afr/Ken-856/2011	KC578635
Ken-857	Kenya	2011	*Miniopterus minor*	BatPV/Min_min/Ken-857/2011	KC578636
Ken-877	Kenya	2011	*Miniopterus minor*	BatPV/Min_min/Ken-877/2011	KC578637
Ken-887	Kenya	2011	*Miniopterus minor*	BatPV/Min_min/Ken-887/2011	KC578638
Ken-898	Kenya	2011	*Miniopterus minor*	BatPV/Min_min/Ken-898/2011	KC578639
Nig-955	Nigeria	2010	*Hipposideros* sp.	BatPV/Hip_sp./Nig-955/2010	KC538903
SA-163	South Africa	2007	*Rhinolophus denti*	BatPV/Rhi_den/RSA-163b/2007	KC578571
SA-170	South Africa	2007	*Eptesicus hottentotus*	BatPV/Ept_hot/RSA-170b/2007	KC578573
SA-172	South Africa	2007	*Rhinolophus denti*	BatPV/Rhi_den/RSA-172/2007	KC578574
SA-724	South Africa	2010	*Neoromicia nana*	BatPV/Neo_nan/RSA-724/2010	KC578578
SA-844	South Africa	2010	*Kerivoula argentata*	BatPV/Ker_age/RSA-844b/2010	KC578580
SA-855	South Africa	2010	*Nycteris thebaica*	BatPV/Nyc_the/RSA-855/2010	KC578581
SA-922	South Africa	2010	*Neoromicia nana*	BatPV/Neo_nan/RSA-922/2010	KC578582
SA-947	South Africa	2010	*Rhinolophus landeri*	BatPV/Rhi_lan/RSA-947/2010	KC578583
SA-1485	South Africa	2012	*Hipposideros caffer*	BatPV/Hip_caf/RSA-1485b/2012	KC578585
SA-1486	South Africa	2012	*Hipposideros caffer*	BatPV/Hip_caf/RSA-1486b/2012	KC578587

Technical Appendix Table 2. Additional paramyxovirus sequences used in phylogenetic analysis

Sequence	Host	Accession number
General		
Avian paramyxovirus 6	Birds	NC_003043
Goose paramyxovirus SF02	Geese	NC_005036
Newcastle disease virus	Birds	NC_002617
Canine distemper virus	Dogs	NC_001921
Feline morbillivirus	Cats	JQ411014
Measles virus	Humans	NC_001498
Peste-des-petits-ruminants virus	Sheep, goats	NC_006383
Rinderpest virus	Cattle	NC_006296
Bovine parainfluenza virus 3	Cattle	NC_002161
Human parainfluenza virus 1	Humans	NC_003461
Sendai virus	Mice, hamsters, rats	NC_001552
Mumps virus	Humans	NC_002200
Porcine rubulavirus	Pigs	NC_009640
Simian virus 41	Monkeys, apes	NC_006428
Fer-de-Lance virus	Reptiles	NC_005084
Beilong virus	Rats	NC_007803
J-virus	Rats	NC_007454
Mossman virus	Rats	NC_005339
Nariva virus	Rats	FJ362497
Tupaia paramyxovirus	Shrews	NC_002199
Bat associated		
Hendra virus	*Pteropus* spp.	NC_001906
Nipah virus	*Pteropus* spp.	NC_002728
Cedar virus	*Pteropus* sp.	JQ001776
Mapuera virus	*Sturnira lilium*	NC_009489
Menangle virus	*Pteropus alecto*	NC_007620
Tioman virus	*Pteropus* spp.	NC_004074
Tuhoko virus 1	*Rousettus leschenaultii*	GU128080
Tuhoko virus 2	*Rousettus leschenaultii*	GU128081
Tuhoko virus 3	*Rousettus leschenaultii*	GU128082
BatPV Hyp_mon/CO2569/CON/2006	*Hypsignathus monstrosus*	HQ660119
BatPMV Pte_par/KCR245M/CR/2010	*Pteronotus parnellii*	JF828295
BatPMV Pte_par/KCR370/CR/2010	*Pteronotus parnellii*	JF828296
BatPV Car_bre/BR100/BRA/2009	*Carollia brevicauda*	HQ660192
BatPV Car_bre/BR102/BRA/2009	*Carollia brevicauda*	HQ660193
BatPV Car_bre/BR96/BRA/2009	*Carollia brevicauda*	HQ660191
BatPV Car_per/BR310/BRA/2009	*Carollia perspicillata*	HQ660194
BatPV Des_rot/BR21/BRA/2008	*Desmodus rotundus*	HQ660187
BatPV Des_rot/BR22/BRA/2008	*Desmodus rotundus*	HQ660188
BatPV Des_rot/BR222/BRA/2009	*Desmodus rotundus*	HQ660189
BatPV Glo_sor/BR190/BRA/2009	*Glossophaga soricina*	HQ660190
BatPV Myo_alc/3–320/BGR/2009	*Myotis alcathoe*	HQ660163
BatPV Myo_bec/NM98–46/GER/2008	*Myotis bechsteinii*	HQ660170
BatPV Myo_dau/NMS09–48/GER/2009	*Myotis daubentonii*	HQ660165
BatPV Myo_myo/N78–14/GER/2008	*Myotis myotis*	HQ660166
BatPV Myo_myo/N78–16/GER/2008	*Myotis myotis*	HQ660167
BatPV Myo_mys/NM98–140/GER/2008	*Myotis mystacinus*	HQ660168
BatPV Myo_mys/NM98–44/GER/2008	*Myotis mystacinus*	HQ660169
BatPV Myo_mys/NM98–47/GER/2008	*Myotis mystacinus*	HQ660171
BatPV Pte_par/KCR245H/CR/2010	*Pteronotus parnellii*	JF828297
BatPV/Col_afr/GB09478/GAB/2009	*Coleura afra*	HQ660155
BatPV/Eid_hel/CD287/DRC/2009	*Eidolon helvum*	HQ660123
BatPV/Eid_hel/CD291/DRC/2009	*Eidolon helvum*	HQ660124
BatPV/Eid_hel/CD297/DRC/2009	*Eidolon helvum*	HQ660125
BatPV/Eid_hel/GB1237/GAB/2005	*Eidolon helvum*	HQ660140
BatPV/Eid_hel/GB1535/GAB/2005	*Eidolon helvum*	HQ660141
BatPV/Eid_hel/GB1659/GAB/2005	*Eidolon helvum*	HQ660142
BatPV/Eid_hel/GB1661-RMH/GAB/2005	*Eidolon helvum*	HQ660143
BatPV/Eid_hel/GB1678/GAB/2005	*Eidolon helvum*	HQ660144
BatPV/Eid_hel/GB3384/GAB/2006	*Eidolon helvum*	HQ660146
BatPV/Eid_hel/GH48/GHA/2008	*Eidolon helvum*	FJ609194
BatPV/Eid_hel/GH-M2/GHA/2009	*Eidolon helvum*	FJ971936
BatPV/Eid_hel/GH-M28/GHA/2009	*Eidolon helvum*	HQ660147
BatPV/Eid_hel/GH-M3/GHA/2009	*Eidolon helvum*	FJ971937
BatPV/Eid_hel/GH-M33/GHA/2009	*Eidolon helvum*	HQ660148
BatPV/Eid_hel/GH-M43/GHA/2010	*Eidolon helvum*	HQ660127
BatPV/Eid_hel/GH-M51a/GHA/2009	*Eidolon helvum*	HQ660132
BatPV/Eid_hel/GH-M6/2009	*Eidolon helvum*	FJ971938
BatPV/Eid_hel/GH-M61a/GHA/2009	*Eidolon helvum*	HQ660133
BatPV/Eid_hel/GH-M63a/GHA/2009	*Eidolon helvum*	HQ660136

Sequence	Host	Accession number
BatPV/Eid_hel/GH-M67a/GHA/2009	*Eidolon helvum*	HQ660131
BatPV/Eid_hel/GH-M69a/GHA/2009	*Eidolon helvum*	HQ660135
BatPV/Eid_hel/GH-M74a/GHA/2009	*Eidolon helvum*	HQ660129
BatPV/Eid_hel/GH-M77/GHA/2009	*Eidolon helvum*	HQ660130
BatPV/Eid_hel/GH-M90a/GHA/2009	*Eidolon helvum*	HQ660134
BatPV/Eid_hel/RCA-P05/RCA/2008	*Eidolon helvum*	HQ660150
BatPV/Eid_hel/RCA-P09/RCA/2008	*Eidolon helvum*	HQ660151
BatPV/Eid-hel/RCA-P10/RCA/2008	*Eidolon helvum*	HQ660149
BatPV/Epo_gam/CD078/DRC/2009	*Epomophorus gambianus*	HQ660128
BatPV/Epo_gam/CD255/DRC/2009	*Epomophorus gambianus*	HQ660120
BatPV/Epo_gam/CD273/DRC/2009	*Epomophorus gambianus*	HQ660122
BatPV/Epo_spe/CD256/DRC/2009	*Epomophorus* sp.	HQ660121
BatPV/Hip_aba/GB59–59/GHA/2009	*Hipposideros abae*	HQ660162
BatPV/Hip_caf/GB09670/GAB/2009	*Hipposideros caffer*	HQ660156
BatPV/Hip_caf/GB09790/GAB/2009	*Hipposideros caffer*	HQ660158
BatPV/Hip_caf/GB59–30/GHA/2009	*Hipposideros caffer*	HQ660161
BatPV/Hip_gig/GB09682/GAB/2009	*Hipposideros gigas*	HQ660157
BatPV/Hip_gig/GB09898/GAB/2009	*Hipposideros gigas*	HQ660159
BatPV/Hip_rub/GB19-S/GHA/2009	*Hipposideros ruber*	HQ660160
BatPV/Hip_rub/GH19–140/GHA/2009	*Hipposideros ruber*	HQ660153
BatPV/Hip_spec/GH19-T/GHA/2009	*Hipposideros* sp.	HQ660154
BatPV/Hyp_mon/RCA-P18/RCA/2008	*Hypsignathus monstrosus*	HQ660152
BatPV/Myo.mys/E20b/09	*Myotis mystacinus*	JN086953
BatPV/Myo_tor/CD356/DRC/2009	*Myonycteris torquata*	HQ660126
BatPV/Myo_tor/CO2225/CON/2005	*Myonycteris torquata*	HQ660118
BatPV/Myo_tor/GB1386/GAB/2005	*Myonycteris torquata*	HQ660137
BatPV/Pip.pip/E95b/09	*Pipistrellus pipistrellus*	JN086954
BatPV/Pip_nan/GH36/GHA/2008	*Pipistrellus nanus*	FJ609192
BatPV/Rou_aeg/GB1583/GAB/2005	*Rousettus aegyptiacus*	HQ660138
BatPV/Rou_aeg/GB1590/GAB/2005	*Rousettus aegyptiacus*	HQ660139
BatPV/Rou_aeg/GB2009/GAB/2005	*Rousettus aegyptiacus*	HQ660145
Eidolon helvum PMV U32A	*Eidolon helvum*	JN862594
Eidolon helvum PMV U42A	*Eidolon helvum*	JN862566
Eidolon helvum PMV U42B	*Eidolon helvum*	JN862577
Eidolon helvum PMV U45A	*Eidolon helvum*	JN862582
Eidolon helvum PMV U45B	*Eidolon helvum*	JN862587
Eidolon helvum PMV U49B	*Eidolon helvum*	JN862572
Eidolon helvum PMV U50B	*Eidolon helvum*	JN862580
Eidolon helvum PMV U50C	*Eidolon helvum*	JN862588
Eidolon helvum PMV U51A	*Eidolon helvum*	JN862564
Eidolon helvum PMV U51B	*Eidolon helvum*	JN862573
Eidolon helvum PMV U53A	*Eidolon helvum*	JN862568
Eidolon helvum PMV U53B	*Eidolon helvum*	JN862569
Eidolon helvum PMV U54A	*Eidolon helvum*	JN862563
Eidolon helvum PMV U54B	*Eidolon helvum*	JN862571
Eidolon helvum PMV U58B	*Eidolon helvum*	JN862583
Eidolon helvum PMV U59A	*Eidolon helvum*	JN862570
Eidolon helvum PMV U59B	*Eidolon helvum*	JN862579
Eidolon helvum PMV U5A	*Eidolon helvum*	JN862592
Eidolon helvum PMV U5B	*Eidolon helvum*	JN862591
Eidolon helvum PMV U5C	*Eidolon helvum*	JN862593
Eidolon helvum PMV U61A	*Eidolon helvum*	JN862578
Eidolon helvum PMV U62A	*Eidolon helvum*	JN862567
Eidolon helvum PMV U63A	*Eidolon helvum*	JN862574
Eidolon helvum PMV U64A	*Eidolon helvum*	JN862575
Eidolon helvum PMV U66A	*Eidolon helvum*	JN862581
Eidolon helvum PMV U66B	*Eidolon helvum*	JN862589
Eidolon helvum PMV U68A	*Eidolon helvum*	JN862584
Eidolon helvum PMV U68B	*Eidolon helvum*	JN862590
Eidolon helvum PMV U6A	*Eidolon helvum*	JN862565
Eidolon helvum PMV U6B	*Eidolon helvum*	JN862562
Eidolon helvum PMV U71A	*Eidolon helvum*	JN862585
Eidolon helvum PMV U71B	*Eidolon helvum*	JN862586
Eidolon helvum PMV U72A	*Eidolon helvum*	JN862576
Eidolon PMV_RC09_216s	*Eidolon helvum*	HE647824
Eidolon PMV_RC09_222k	*Eidolon helvum*	HE647825
Eidolon PMV_RC09_236s2	*Eidolon helvum*	HE801056
Miniopterus griveaudi PMV SMG16723 1	*Miniopterus griveaudi*	JQ886099
Miniopterus griveaudi PMV SMG16723 2	*Miniopterus griveaudi*	JQ886100
Miniopterus griveaudi PMV SMG16753 1	*Miniopterus griveaudi*	JQ886101
Miniopterus griveaudi PMV SMG16753 2	*Miniopterus griveaudi*	JQ886102
Miniopterus griveaudi PMV SMG16756	*Miniopterus griveaudi*	JQ886103
Miniopterus sororculus PMV SMG16797	*Miniopterus sororculus*	JQ886104

Sequence	Host	Accession number
Mormopterus acetabulosus PMV SMG17000	*Mormopterus acetabulosus*	JQ886105
Paramyxovirus bat/GH10/GHA/2008	*Eidolon helvum*	FJ609191
Paramyxovirus bat/GH15/GHA/2009	*Eidolon helvum*	FJ971935
Paramyxovirus bat/GH21a/GHA/2009	*Eidolon helvum*	FJ971939
Paramyxovirus bat/GH27a/GHA/2009	*Eidolon helvum*	FJ971940
Paramyxovirus IFBPV01/2010	*Pteropus vampyrus*	AB748561
Paramyxovirus IFBPV32/2011	*Pteropus* sp.	AB748559
Paramyxovirus IFBPV46/2011	*Pteropus* sp.	AB748560
Triaenops menamena PMV SMG16462	*Triaenops menamena*	JQ886096
Triaenops menamena PMV SMG16505	*Triaenops menamena*	JQ886098
